# Supplementary material for: FastqPuri: high-performance preprocessing of RNA-seq data
Source: BMC Bioinformatics. 2019 May 3;20:226. doi: 10.1186/s12859-019-2799-0 (PMC6500068; doi:10.1186/s12859-019-2799-0)
Supplement: Supplementary file 2 — Archive of FastqPuri. Archive containing all files needed to install and run FastqPuri v1.0.6. Date stamp March 22, 2019. (GZ 47,819 kb) [file 12859_2019_2799_MOESM2_ESM.gz › FastqPuri-1.0.6/html/md_PANDOC.html]

My Project: Installing Pandoc


My Project

Installing Pandoc

#### Windows and Mac OS X

The pandoc installation page includes easy to use installers for Windows and Mac OS X.

#### Linux

The version of pandoc included in the standard repositories is not recent enough for use with the **rmarkdown** package. You can install a more recent version of pandoc by installing the Haskell Platform and then following these instructions for building pandoc from source.

This method installs a large number of Haskell dependencies so might not be desirable. You can also obtain a standalone version of pandoc without the dependencies as follows:

##### Older Systems (RHEL 5)

For older Linux systems you can obtain a standalone version of pandoc v1.13.1 (with no Haskell dependencies) from https://copr.fedoraproject.org/coprs/petersen/pandoc-el5/ as follows:

```
$ sudo wget -P /etc/yum.repos.d/ https://copr.fedoraproject.org/coprs/petersen/pandoc-el5/repo/epel-5/petersen-pandoc-el5-epel-5.repo
$ yum install pandoc pandoc-citeproc
```

##### Newer Systems (Debian/Ubuntu/Fedora/RHEL6+)

For newer Linux systems you can make a standalone version of pandoc v1.12.3 available to the system by soft-linking the binaries included with RStudio:

```
$ sudo ln -s /usr/lib/rstudio/bin/pandoc/pandoc /usr/local/bin
$ sudo ln -s /usr/lib/rstudio/bin/pandoc/pandoc-citeproc /usr/local/bin
```

If you are running RStudio Server the commands would be:

```
$ sudo ln -s /usr/lib/rstudio-server/bin/pandoc/pandoc /usr/local/bin
$ sudo ln -s /usr/lib/rstudio-server/bin/pandoc/pandoc-citeproc /usr/local/bin
```

If you aren't running RStudio at all you can simply copy the binaries out of the RStudio `bin/pandoc` directory and locate them within `/usr/local/bin` on your target system.


---

Generated by  

 1.8.14
